# Prevalence of dental caries in a pediatric population during the COVID-19 omicron pandemic era in South India

**DOI:** 10.3205/dgkh000466

**Published:** 2024-03-05

**Authors:** Manikandan Gunasekaran, Karthik Shunmugavelu, Kavitha Ponnusamy, Aditya Shinde, Selvam Azhagarsamy, Shobana Murali

**Affiliations:** 1 Dept of Dentistry, Shri Sathya Sai Medical College and Research Institute, Tamilnadu, India; 2 PSP Medical College Hospital and Research Institute Tambaram Kanchipuram, Tamilnadu, India; 3 Government Omandurar Medical College, Chennai, Tamilnadu, India; 4 MGM Dental College, Navi Mumbai, India; 5 Department of Dental surgery, Government Theni medical college, Theni, Tamilnadu, India; 6 Ragas Dental College and Hospital, Chennai, Tamilnadu, India

**Keywords:** dental caries, pediatric population, SARS-CoV-2, Covid-19, Omicron, South India

## Abstract

Caries is a multifactorial disease that involves a majority of the pediatric population. If not diagnosed and treated, it can lead to severe consequences affecting the permanent dentition. The objective of this study was to assess the prevalence of oral foci of infection in a multispeciality hospital during the pandemic in Chennai, South India. The majority of the patients examined had caries.

## Abstract (de)

Karies ist eine multifaktorielle Erkrankung, von der ein Großteil der pädiatrischen Bevölkerung betroffen ist. Wenn sie nicht diagnostiziert und behandelt wird, kann sie schwerwiegende Folgen für das bleibende Gebiss haben. Ziel dieser Studie ist es, die Prävalenz oraler Infektionsherde in einem multidisziplinären Krankenhaus während der Pandemie in Chennai, Südindien, zu untersuchen. Die Mehrheit der untersuchten Patienten hatte Zahnkaries.

## Introduction

Caries is a multifactorial, biofilm-mediated, diet modulated, non-communicable and dynamic disease that results in net mineral loss of dental hard tissues [1]. “Dental caries” originates from the Latin word “caries” which means decay. It is primarily associated with *Streptococcus* *mutans*, which demineralizes the hard dental tissues [2]. The most commonly accepted theory for the disease etiology is the ‘chemo-parasitic theory’ proposed by W. D. Miller in 1891 [3]. He suggested that the combined effects of acids and the oral microorganisms results in tooth decalcification [4], [5].

Approximately 2.4 billion people world wide have permanent teeth affected by dental caries. More than 530 million members of pediatric population lose their primary teeth due to dental caries per year [5], [6], [7], [8]. 

In children, the prevalence of dental caries is affected by several factors. including socio economic status, inappropriate dietary factors and altered immune response. This article aimed to assess prevalence of dental caries at a multispeciality hospital during the pandemic in Chennai, South India. Treatments involving dental caries management were also assessed. 

## Materials and methods

This study was performed from 2020–2021. A total of 257 pediatric patients were assessed. Demographics, chief complaint, past dental history, past medical history, results of extra-oral and intra-oral examinations were documented. The intraoral examination assessed hard tissue, soft tissue and radiographs. Dental caries was diagnosed based on the clinical examination and radiographs. The DMFT/dmft index was used to assess the caries status. Caries disease indicators such as visible cavitations, active white-spot lesions, interproximal radiographic lesions penetrating to the dentin, and a history of any cavitations were also assessed. An explorer which catches or resists removal when moderate pressure is applied was used as a clinical indicator of dental caries. Caries management was also assessed. Convenience sampling is a limitation to this study, as is data acquisiton on in Chennai. The other limitation includes lack of a proper diagnostic scale. The results were collected and analysed with descriptive statistics using the Statistical Package for Social Sciences (SPSS version 2).

## Results

### Demographics

The gender distribution was 51% female and 49% male.

### Prevalence of dental caries

The caries prevalence was 82.1%. Out of 257 patients examined, 211 patients presented with dental caries.

### Site of the lesion

Most of the carious lesions were located on the occlusal surface (65%), followed by the incisal (24%) and distal surfaces (11%).

### Management of dental caries

The majority of the lesions were treated by restorations (76%) followed by extractions (20%), prosthetic replacement (3%) and orthodontic treatment (1%)

## Discussion

Out of the 257 paediatric patients examined, 211 presented with dental caries. There was no significant difference in the gender distribution. The diagnosis of carious lesions was primarily a visual process, based principally on clinical inspection and review of radiographs. Tactile information obtained through use of the dental explorer or “probe” was used in the diagnostic process. A majority of the diagnosed carious lesionswas present on the occlusal surfaces, followed by the incisal and proximal surfaces. This can be attributed to improper brushing technique, poor oral hygiene and inappropriate diet, such as sucking on toffees and other candy. The majority of the lesions were treated by restorations. However, extractions were also carried, out followed by prosthetic replacement. Orthodontic treatment was done in only one of the pediatric patients. 

## Conclusion

Dental caries one of the most common dental problems in toddlers and children. If not diagnosed and treated, it can lead to severe consequences that will also the permanent dentition. The importance of fluoridation and proper oral hygiene technique must be taught.

## Notes

### Competing interests

The authors declare that they have no competing interests.

### Author’s ORCID

Karthik Shunmugavelu: 0000-0001-7562-8802
